# Predictors for successful psychotherapy: Does migration status matter?

**DOI:** 10.1371/journal.pone.0257387

**Published:** 2021-09-16

**Authors:** Friederike Kobel, Yesim Erim, Eva Morawa

**Affiliations:** Department of Psychosomatic Medicine and Psychotherapy, University Hospital Erlangen, Friedrich-Alexander University Erlangen-Nürnberg (FAU), Erlangen, Germany; Medical University of Vienna, AUSTRIA

## Abstract

**Background:**

We investigated, if migration status, and additional sociodemographic and clinical factors, are associated with somatization and depressiveness at admission and with remission after inpatient psychotherapy.

**Methods:**

Multiple linear and binary logistic regression analyses were used to identify predictors for severity of somatoform and depressive symptoms at admission of inpatient psychotherapy (T0), and for remission after inpatient psychotherapy (T1). We tested the association between symptoms concerning somatization (PHQ-15: Patient-Health-Questionnaire Somatization Module) and depression (PHQ-9: Patient-Health-Questionnaire Depression Module) and several sociodemographic and clinical factors in 263 patients at admission. For remission after treatment, we additionally included severity of symptoms at admission, number of diagnoses and duration of treatment in the regression models. Remission after treatment was defined as response plus a post value of less than 10 points in the respective questionnaire. Clinical relevance was interpreted using effect sizes (regression coefficients, Odds Ratio (OR)) and Confidence Intervals (CI).

**Findings:**

Significant and clinically relevant predictors for high symptom severity at T0 were lower education (β = -0.13, p = 0.04), pretreatment(s) (β = 0.205, p = 0.002) and migration status (β = 0.139, p = 0.023) for somatization, and potential clinically relevant predictors (|β|>0.1) for depression were living alone (β = -0.116, p = 0.083), pretreatment(s) (β = 0.118, p = 0.071) and migration status (β = 0.113, p = 0.069). At T1 patients with pretreatment(s) (OR = 0.284 [95% CI: 0.144, 0.560], p<0.001) and multiple diagnoses (OR = 0.678 [95% CI: 0.472, 0.973], p = 0.035) were significantly and clinically relevant less likely to show a remission of depressive symptoms. In addition, a potentially clinically meaningful effect of migration status on remission of depressive symptoms (OR = 0.562 [95% CI: 0.264, 1.198], p = 0.136) cannot be ruled out. For somatoform symptoms pretreatment(s) (OR = 0.403, [95% CI: 0.156, 1.041], p = 0.061) and education (OR = 1.603, [95% CI: 0.670, 3.839], p = 0.289) may be regarded as clinically relevant predictors for remission.

**Conclusion:**

The results of our study suggest that migration status has a clinically relevant influence on severity of somatoform and depressive symptoms at admission. Clinical relevance of migration status can also be assumed regarding the remission of depression. Migration status and further factors affecting the effectiveness of the treatment should be analyzed in future research among larger samples with sufficient power to replicate these findings.

## Introduction

Inpatient psychotherapy is crucial for the treatment of patients who suffer from psychological disorders and yet, investigation on predictors for successful psychotherapy is scarce. In Germany inpatient psychotherapy is mostly embedded in a multimodal treatment plan in psychosomatic departments.

When an indication is secured, multimodal and multicomponent inpatient psychotherapy is free of charge for all insured patients in Germany. Inpatient psychotherapy is based on depth psychological or behavioral methods and includes, besides individual and group psychotherapy, several other methods such as psychoeducation, medical treatment, family sessions, body- and art therapy. Specialized nurses give supportive interventions and skills training [1]. The vast majority of studies in inpatient psychosomatic settings has been conducted in Germany [2–5], since here inpatient psychotherapy is nationwide supplied as a regular health insurance service.

### Migrants in Germany

In 2005 the term “migratory background” was first introduced officially into German statistics [6]. The German Poll “Mikrozensus”, a representative population-based survey run yearly by the German National Institute of Statistics, indicated in 2005 that 18.6% of the population had a “migratory background”. This number increased to 26.0% in 2019 [7]. In the meantime, a new term, in order to substitute the term “migratory background”, was proposed by the Federal Government Expert Commission on the framework for sustainable integration (Fachkommission für Integrationsfähigkeit): immigrants and their direct descendants or correspondingly first- and second-generation immigrants [8]. In contrary to the first article of this study we will now use this new term instead (belonging to first- or second-generation immigrants). Equally to the former concept “migratory background”, it defines a person as an immigrant or their direct descendant (first and second generation) if he/she or at least one of his/her parents did not obtain German citizenship by birth. This includes immigrant and nonimmigrant foreigners, immigrant, and nonimmigrant naturalized persons, (late) emigrants ((Spät-) Aussiedler), persons who obtained German citizenship through adoption, and German-born children from the above-mentioned groups [9].

With 13.3% persons of Turkish origin constitute the largest group, followed by persons of Polish (10.5%), Russian (6.5%), Romanian (4.8%), and Italian (4.1%) origin [7]. In view of the steady increase (18.6% 2005 to 26.0% 2019) of this population group and of the ongoing diversification of the origin and reason of migration (wars, armed conflicts, lack of economic perspective, effects of climate change), the investigation of direct or indirect migration experience, as a potential influencer of mental health concerning psychotherapy, is gaining more importance.

### Predictors for symptom severity at baseline

Several studies showed that the level of symptom severity at admission influences the effectiveness of psychotherapy [10, 11]. Therefore, it is necessary to investigate which factors have an impact on symptom severity at admission. Among environmental characteristics which influence mental health, a low socioeconomic status is one of the most important factors connected to poorer mental health [12–14]. Regarding the association between migration and mental health existing evidence is inconsistent. While most German studies find migrant patients to have more symptom burden concerning psychological disorders at the beginning of inpatient psychotherapy [15–20], international studies show a more heterogenous picture [21–23]. Wiborg et al. suggest that belonging to first- or second-generation immigrants is a significant predictor for high symptom burden at admission and for worse treatment outcome [24]. Furthermore, they detected that previous psychotherapeutic treatment predicted a lower level of symptom severity at baseline, whereas suicidality, high levels of posttraumatic stress and interpersonal problems were observed as significant predictors for high symptom burden at baseline. Studies from German population-based samples showed that among Turkish immigrants the female gender is an important predictor for more symptoms of somatization [25]. Pre- (i.e., living conditions in the country of origin, traumatization) and post-migratory stress (i.e., acculturative stress, perceived discrimination, lower socioeconomic status) can presumably account for these discrepancies [7, 26–29]. Several studies showed that, based on different acculturative strategies [30], integration or assimilation are associated with better mental health whereas marginalization is associated with poorer mental health [27, 31, 32]. A very important post-migratory stressor seems to be (perceived) discrimination in the host country. In studies from different countries all over the world it is shown that discrimination can lead to poorer mental health in immigrants [21, 26, 33–37]. In line with findings on the role of lower socioeconomic status on mental health of non-migrants, this factor also seems to negatively influence the mental health of immigrants [34].

At the same time, other studies support the “healthy-migrant hypothesis”, stating that mainly people in good health conditions migrate to other countries [23, 38, 39]. Salas-Wright et al. [38] demonstrated that immigrants had less probability for a lifetime disorder (Adjusted OR = 0.63, 95% CI = 0.57–0.71), and were less likely to derive from families with a history of mental health problems than US-natives. The prevalence of mental disorders did not differ significantly between immigrant children and US-born individuals. Immigrants who migrated as adolescents or adults, however, had a significantly lower psychiatric morbidity which would confirm the healthy-migrant theory. Some studies demonstrate that, especially for children and adolescents belonging to first- or second-generation immigrants, good bicultural competencies can have a positive effect on their mental health [40, 41]. A large population-based representative survey from Canada showed that recently arrived migrants showed better mental health than “long-term” migrants. However, this effect attenuated in duration of residence [42].

In conclusion current data on the topic of mental health differences between immigrants and non-immigrants shows inconsistencies. Considering the results from German studies we hypothesize that patients belonging to first- or second-generation immigrants show higher psychological symptom burden than autochthone patients. Yet it is important to enhance that the migration status per se does not lead to poorer mental health. More precise it is the dynamic process of migration with its pre- and post-migratory stressors which has various implications on the mental health of immigrants.

### Migratory background as a predictor for remission

Mösko et al. indicated that the Turkish origin constitutes an independent negative predictor for psychotherapeutic treatment outcome in inpatient rehabilitation [15]. In addition, they analyzed predictors for treatment outcome separately for patients with and without Turkish origin. Apart from high symptom severity at admission as a common predictor, the groups had different predictors for treatment outcome. Among patients with Turkish origin significant negative predictors for treatment outcome were the duration of unemployment and the presence of somatoform and stress- and adjustment disorders. Other studies underlined that the migration status is a significant negative predictor for psychotherapeutic treatment outcome [20, 43].

The question of how these discrepancies can be explained is still under investigation. In general, it can be assumed that personal and environmental resources such as social support, illness beliefs, motivation towards psychotherapy and the therapeutic relationship are important for psychotherapeutic treatment success [44–46]. Studies have shown that illness beliefs vary widely among different cultures [47–50], psychotherapy is not known or attitudes towards it are more skeptical among migrant patients [51–54]. Presumably the forementioned pre- and post-migration stressors can also add to less remission for migrant patients than for non-migrant patients.

### General predictors for remission

Previous research concerning the predictors of psychotherapeutic treatment outcome in general showed that several sociodemographic and clinical factors influence the remission after psychotherapy. Rehabilitation studies showed that clinical factors, such as long treatment duration and high symptom severity at admission, represent positive predictors for treatment outcome. Whereas low educational degree, long duration of disease, diagnosis of somatoform disorders, long duration of unemployment and personality disorders are negative predictors for treatment outcome [15, 55]. Further negative predictors for psychotherapeutic treatment outcome are for example: psychiatric comorbidities, lower motivation towards psychotherapy and the absence of a permanent relationship [56, 57]. Further positive predictors for symptom improvement during psychotherapy were found to be an early improvement after psychotherapy onset, the full completion of psychotherapeutic treatment, paid employment as an important sociodemographic predictor, higher symptom burden at admission, the absence of personality disorders and a better subjective quality of life [10, 58, 59].

Taking previous research on the topic into consideration we aimed to examine whether:

1. belonging to first- or second-generation immigrants is an independent predictor for the severity of depressive and somatoform symptoms at the beginning of inpatient psychotherapy?
2. there are other predictors for the severity of depressive and somatoform symptoms at the beginning of inpatient psychotherapy?
3. belonging to first- or second-generation immigrants is an independent predictor for symptom remission at the end of inpatient psychotherapy?
4. there are other predictors for symptom remission at the end of inpatient psychotherapy?

## Materials and methods

### Ethical standards

The present study was approved by the Ethics Committee of the Medical Faculty of the Friedrich-Alexander University Erlangen-Nürnberg (FAU) (Project identification code: 232_14B). Written informed consent was obtained from all participants.

### Design and procedure

Data acquisition took place between October 2018 and October 2019 at the inpatient unit and day clinic of the University Department of Psychosomatic Medicine and Psychotherapy in Erlangen and its affiliated Psychosomatic Department at the Community Hospital of Ebermannstadt. An indication for inpatient psychotherapy is given when outpatient psychotherapy cannot sufficiently treat symptoms. Inclusion criteria for the study were being off age, no acute psychotic disorder or acute suicidality, and sufficient German knowledge to understand and answer the questionnaires. If informed consent was given, patients were enrolled in the study at admission (T0), if the date of questionnaire completion did not go beyond the admission date by more than 10 days and, if at least 50% of the questionnaires were filled out. Patients were enrolled at discharge (T1) if completion of the questionnaire and the discharge date did not differ by more than 10 days and if they had undergone at least 28 days of treatment. Treatment offered was equal in both departments. Every patient received the same treatment schedule in both departments. The departments under investigation follow an integrative psychotherapeutic multimodal and multiprofessional approach (in single and group therapy), including integrative depth-psychological and behavioral therapeutic concepts. Additionally, the clinics offer psychoeducation, medical treatment, interaction groups, disorder-specific group therapy, depth-psychologically based movement and art therapy, skills training (M. Linehan), mindfulness practice, and diagnostic family sessions are delivered. All treatments are performed in German for all patients. The duration of treatment was by default eight weeks but could have been extended for maximum two weeks depending on the individual situation. Within one week after admission and at least one week before discharge patients were asked to answer the questionnaires. International Statistical Classification of Diseases and Related Health Problems, 10th revision (ICD-10)- coded diagnoses (F-diagnoses) were extracted from the therapists’ letters at discharge. We defined immigrants and their direct descendants (first- and second-generation immigrants) using the definition of “migratory background” from the German Poll “Mikrozensus” in 2017 [9]. The clinics’ therapists are regularly trained and supervised regarding a culturally sensitive approach in therapy. Furthermore, they are trained to include the patients’ history of migration in their anamnesis and address migratory issues during therapy. The effectiveness of inpatient psychotherapy was dealt with in detail in the first article of this study [60].

### Instruments

#### Patient Health Questionnaire: Somatization Module (PHQ-15)

The Patient Health Questionnaire (PHQ) is an established self-assessment screening instrument for common mental disorders. The somatization module (PHQ-15) has 13 items and is used to diagnose somatoform disorders and to grade somatic complaints. Answers range from 0 (“not bothered at all”) to 2 (“bothered a lot”). Item 14 and 15 coincide with the PHQ-9. Cut-off values of 5, 10, and 15 serve to differentiate mild, moderate, and severe symptom levels, respectively [61]. In the validation study, Cronbach’s α was 0.80 [62]. In the German validation study, Cronbach’s α was found to be 0.79 [63]. In the present study, we obtained an internal consistency (Cronbach’s α) of 0.81 at T0 and 0.82 at T1. The questionnaire contains one item which is gender-specific for women (pain during menstruation or other menstruation problems). Women can therefore score higher than men. To equalize this condition, we calculated without the menstruation item (PHQ-15*).

#### Patient Health Questionnaire: Depression Module (PHQ-9)

The PHQ-9 serves to measure the severity of depressive symptoms and to categorize patients with major depression. It is aligned with nine main criteria to diagnose major depression [64] and has nine items that can be answered on a scale from 0 (not at all) to 3 (nearly every day). Scores of 0–4, 5–9, 10–14, 15–19, and 20–27 indicate minimal, mild, moderate, moderately severe, and severe depression, respectively [61]. The validation study showed Cronbach’s α = 0.89 [65]. Cronbach’s α in the German validation study was found to be 0.88 [63]. In the present study, Cronbach’s α was 0.84 at T0 and 0.88 at T1.

## Statistical analysis

All analyses were conducted using SPSS V.24. All patients had filled out at least 50% of the questionnaires. After analyzing missing values, questionnaires with ≤20% missing values were completed with the expectation-maximization algorithm. Means and standard deviations, were computed to profile sociodemographic, migration-specific, and clinical characteristics. At discharge only patients who matched inclusion criteria and were under treatment for ≥28 days were included in the remission analysis. For comparisons between groups, we calculated T-tests for metric variables. When normal distribution was not given, nonparametric tests, such as Mann–Whitney U-test for independent variables, were used. To test for differences of categorical variables, chi-squared tests were applied.

Clinical significant changes were analyzed with the reliable change index [66]:

$$\mathrm{RCI}=\frac{x_{\mathrm{post}}-x_{\mathrm{pre}}}{s_{\mathrm{diff}}},\;S_{\mathrm{diff}}=\sqrt{2\times {\left(S_{E}\right)}^{2}},\;S_{E}=\mathrm{SD}\times \sqrt{1-r_{\mathrm{xx}}}$$

where X_post_ is posttest value, X_pre_ is pretest value, S_diff_ is standard error of difference between the two test scores, SD is standard deviation of the norm population, and r_xx_ is Cronbach’s α.

Remission after treatment was defined as response to treatment plus a post value of less than 10 points in the respective questionnaire. More information can be found in the first article of this study [60].

A multiple linear regression analysis with enter method was used to test the association between sociodemographic and clinical factors (age, gender, education, living with partner, pretreatment and belonging to first- or second-generation immigrants) and the symptom severity of depression and somatoform disorders at admission. Similarly, a multiple binary logistic regression analysis was calculated to test the association between the above-mentioned factors, and additionally the number of diagnoses and symptoms at admission, as well as the duration of treatment at discharge and the remission of depressive and somatoform symptoms. Education, pretreatment, and duration of treatment were dichotomized (education: below secondary/ vocational education vs. secondary/ vocational education or university degree, pretreatment: none vs. ≥ 1, duration of treatment: ≤ 8 weeks vs. >8 weeks). In all analyses, a significance level of p ≤ 0.05 was determined. In line with the CONSORT 2010 guidelines [67] clinical relevance was interpreted using effect sizes (regression coefficients and odds ratio, OR) and CI. In case of multiple linear regression analysis, a predictor was considered clinically relevant when |β| >0.1. Regarding multiple binary regression analysis, predictors were considered clinically significant when odds ratio (OR) >1.5 (for OR >1) and OR <0.67 (for OR <1) [68].

## Results

### Sociodemographic characteristics

Of 328 patients which entered treatment during the period of acquisition, 48 were non responders. Of 280 we had to apply exclusion criteria for 17 patients. At discharge 229 patients were counted as completers and therefore were included at remission analysis. For more information see: Kobel et al. [60]. Among these we included 55 first- or second-generation immigrants at admission, and at discharge 48 were included in the analysis of PHQ-9 and 47 of PHQ-15. More information can be found in **Table 1**.

**Table 1:** Sociodemographic data of the total sample and patients with and without migration status.

	Total sample (N = 263)	Native German patients (n = 208)	Immigrant patients# (n = 55)
**Gender, n (%)**			
Women	180 (68.4)	139 (66.8)	41 (74.5)
Men	83 (31.6)	69 (33.2)	14 (25.5)
**Age (years)**			
M (SD)	39.3 (13.3)	39.3 (13.7)	39.5 (11.7)
**Living together with partner, n (%)**			
Yes	124 (47.1)	96 (46.2)	28 (50.9)
No	134 (51.0)	107 (51.4)	27 (49.1)
No data	5 (1.9)	5 (2.4)	-
**Education, n (%)**			
Below secondary/ vocational education^a^	161 (61.2)	127 (61.1)	34 (61.8)
Secondary/ vocational and higher education^b^	99 (37.6)	79 (38.0)	20 (36.4)
No data	3 (1.1)	2 (1.0)	1 (1.8)
**Employment status, n (%)**			
Unemployed^c^	85 (32.3)	69 (33.2)	16 (29.1)
Employed^d^	174 (66.2)	137 (65.9)	37 (67.3)
No data	4 (1.5)	2 (1.0)	2 (3.6)
**Duration of treatment (days)**			
M (SD)	55.3 (16.5)	55.3 (18.0)	55.3 (8.2)
**Number of pretreatments** ^**e**^ **n (%)**			
None	126 (47.9)	96 (46.2)	30 (53.4)
≥1	136 (51.7)	111 (53.4)	25 (45.5)
No data	1 (0.4)	1 (0.5)	-
**Number of diagnoses** ^**f**^ **, n (%)**			
one	34 (12.9)	24 (11.5)	10 (18.2)
two	109 (41.4)	89 (42.8)	20 (36.4)
three	80 (30.4)	62 (29.8)	18 (32.7)
four	33 (12.5)	26 (12.5)	7 (12.7)
five	5 (1.9)	5 (2.4)	-
six	2 (0.8)	2 (1.0)	-
**Symptoms at admission (T0)**			
PHQ-9			
M (SD)	14.4 (5.7)	14.1 (5.8)	15.5 (5.3)
PHQ-15*			
M (SD)	12.5 (5.4)	12.2 (5.4)	13.9 (5.4)

# first- or second-generation immigrants

^a^ no educational certificate, primary school, middle school, other

^b^ university degree

^c^ unemployed, job-seeking, pensioner, pension because of a reduction in earning capacity, sick leave, other

^d^ employed full-time, employed part-time, trainee, student

^e^ hospital/ daily hospital psychosomatic/ psychiatric treatments

^f^ number of ICD-10 coded mental and behavioral disorders

* without menstruation item; number of samples may vary

### Migration related characteristics

More than half of migrant patients lived in Germany in the second generation (52.7%), the average length of residence of the first generation was 27.2 years (SD: 10.2). More than two third had the German citizenship (76.4%). Out of the entire migrant sample 41.8% spoke German as their mother tongue, 21.8% indicated very good, 29.1% good and only 7.3% moderate German language proficiency. The most frequent countries of origin were Poland (n = 10, 18.2%) and Turkey (n = 7, 12.7%). For more information see: Kobel et al. [60].

### Migration status: Predictor at baseline?

At baseline belonging to first- or second-generation immigrants was a potential clinically relevant predictor for symptom severity of depression (p = 0.069, β = 0.113). For somatization belonging to first- or second-generation immigrants was a significant predictor in the unadjusted model (p = 0.035, β = 0.131), and remained an independent significant and clinically relevant predictor when controlling for important sociodemographic and clinical factors (p = 0.023, β = 0.139) **(Table 2)**.

**Table 2:** Results of multiple linear regression analysis at admission (T0).

Dependent variable	PHQ-9					PHQ-15*				
	Regression coefficient [95% CI]	Standard error	Beta	T	p	Regression coefficient [95% CI]	Standard error	Beta	T	p
**Unadjusted model**										
Constant	14.095 [13.317, 14.873]	0.395	-	35.667	<0.001	12.169 [11.425, 12.914]	0.378	-	32.207	<0.001
MIG	1.355 [-0.346, 3.057]	0.864	0.097	1.568	0.118	1.746 [0.122, 3.369]	0.824	0.131	2.118	**0.035**
**Adjusted model**										
Constant	14.287 [11.528,17.045]	1.401	-	10.201	<0.001	9.714 [7.095, 12.334]	1.330	-	7.305	<0.001
Age	-0.023 [-0.080, 0.034]	0.029	-0.053	-0.804	0.422	0.033 [-0.021, 0.088]	0.028	0.079	1.197	0.232
Gender	0.777 [-0.725, 2.280]	0.763	0.063	1.019	0.309	0.969 [-0.436, 2.374]	0.713	0.084	1.358	0.176
Education	0.139 [-1.348, 1.625]	0.755	0.012	0.184	0.854	-1.448 [-2.830, -0.067]	0.701	-0.130	-2.065	**0.040**
Living with partner	-1.327 [-2.831, 0.177]	0.764	-0.116	-1.738	0.083	-0.123 [-1.538, 1.291]	0.718	-0.011	-0.172	0.864
Pretreatment	1.359 [-0.119, 2.836]	0.750	0.118	1.811	0.071	2.229 [0.841, 3.616]	0.704	0.205	3.164	**0.002**
Migration status#	1.587 [-0.123, 3.297]	0.868	0.113	1.828	0.069	1.855 [0.259, 3.451]	0.810	0.139	2.289	**0.023**

PHQ-9: Patient Health Questionnaire Depression Module; PHQ-15*: Patient Health Questionnaire Somatization Module without menstruation item; CI: confidence interval; # first- or second-generation immigrants; Independent variables in the regression model: Age; Gender: 0 = male, 1 = female; Education: dichotomized: 0 = no educational certificate, primary school, middle school, other, 1 = secondary/ vocational, university degree; Living with a partner: 0 = no, 1 = yes; Pretreatment: 0 = none, 1≥ 1; MIG: 0 = no, 1 = yes; significant p-values are marked in bold; R^2^ unadjusted model: PHQ-9 = 0.009, PHQ-15 = 0.017; R^2^_adj_ unadjusted model: PHQ-9 = 0.006, PHQ-15 = 0.013; R^2^ adjusted model: PHQ-9 = 0.058, PHQ-15 = 0.102; R^2^_adj_ adjusted model: PHQ-9 = 0.035, PHQ-15 = 0.080.

### General predictors for symptom severity at baseline

Living alone, pretreatment(s) and belonging to first- or second-generation immigrants were potentially clinically meaningful predictors for severity of depression at baseline (p = 0.083, β = -0.116; p = 0.071, β = 0.118; p = 0.069, β = 0.113). For somatization, lower education (p = 0.040, β **=** -0.130), pretreatment(s) (p = 0.002, β **=** 0.205) and belonging to first- or second-generation immigrants (p = 0.023, β **=** 0.139) were significant and clinically relevant predictors for higher symptom severity at baseline. The explanation of variance for the adjusted model at baseline was 5.8% for PHQ-9 and 10.2% for PHQ-15 **(Table 2).**

### Migration status: Predictor for successful psychotherapy?

Belonging to first- or second-generation immigrants can be regarded as clinically relevant for the likelihood of a remission of depressive symptoms (p = 0.136, OR = 0.562, [95% CI: 0.264, 1.198]). **(Table 3)**.

**Table 3:** Results of multiple binary logistic regression analysis for remission at discharge (T1).

Dependent variable	PHQ-9 remission						PHQ-15* remission					
	B	Standard error	Wald	OR	95% CI	p	B	Standard error	Wald	OR	95% CI	p
**Unadjusted model**												
Constant	-0.523	0.154	11.492	0.593	-	0.001	-1.812	0.216	70.521	0.163	-	<0.001
MIG	-0.266	0.347	0.585	0.767	0.388, 1.515	0.444	-0.110	0.487	0.051	0.896	0.345, 2.328	0.821
**Adjusted model**												
Constant	0.002	0.853	0.000	1.002	-	0.998	-2.674	1.172	5.204	0.069	-	0.023
Age	0.021	0.013	2.608	1.021	0.995, 1.048	0.106	0.017	0.019	0.866	1.018	0.981, 1.056	0.352
Gender	-0.066	0.321	0.043	0.936	0.499, 1.756	0.836	0.237	0.451	0.277	1.268	0.524, 3.070	0.599
Education	-0.326	0.329	0.984	0.722	0.379, 1.375	0.321	0.472	0.445	1.123	1.603	0.670, 3.839	0.289
Living with partner	-0.378	0.329	1.322	0.685	0.360, 1.305	0.250	0.323	0.439	0.541	1.381	0.584, 3.267	0.462
Pretreatment	-1.257	0.346	13.214	0.284	0.144, 0.560	**<0.001**	-0.909	0.484	3.523	0.403	0.156, 1.041	0.061
Number of diagnoses	-0.388	0.184	4.440	0.678	0.472, 0.973	**0.035**	-0.370	0.260	2.023	0.691	0.415, 1.150	0.155
Symptoms at admission PHQ-9	0.042	0.028	2.259	1.043	0.987, 1.101	0.133	-	-	-	-	-	-
Symptoms at admission PHQ-15	-	-	-	-	-	-	0.077	0.044	3.101	1.080	0.991, 1.177	0.078
Duration of treatment	-0.164	0.356	0.213	0.849	0.423, 1.704	0.645	0.004	0.471	0.000	1.004	0.399, 2.526	0.994
Migration status#	-0.575	0.386	2.226	0.562	0.264, 1.198	0.136	-0.318	0.512	0.387	0.727	0.267, 1.983	0.534

PHQ-9: Patient Health Questionnaire Depression Module; PHQ-15*: Patient Health Questionnaire Somatization Module without menstruation item; remission: Remission RCI< -1.96 and post value< 10; B: regression coefficient; OR: odds ratio; CI: confidence interval; # first- or second-generation immigrants; Independent variables in the regression model: Age; Gender: 0 = male, 1 = female; Education: dichotomized: 0 = no educational certificate, primary school, middle school, other, 1 = secondary/ vocational, university degree; Living with a partner: 0 = no, 1 = yes; Pretreatment: 0 = none, 1 = ≥1; Number of diagnoses: number of ICD-10 coded mental and behavioral disorders 1 = 1, 2 = 2, 3 = 3, 4 = 4, 5, 6; Symptoms at admission: severity of symptom concerning the respective independent variable; Duration of treatment: 0 = ≤8 weeks, 1 = >8 weeks; MIG: 0 = no, 1 = yes; significant p-values are marked in bold; Nagelkerkes R^2^: Unadjusted model PHQ-9 = 0.004, PHQ-15 = 0.000; Nagelkerkes R^2^: Adjusted model PHQ-9 = 0.159, PHQ-15 = 0.084.

### General predictors for successful psychotherapy

Pretreatment(s) (p < 0.001, OR = 0.284 [95% CI: 0.144, 0.560]) and having more diagnoses (p = 0.0035, OR = 0.678 [95% CI: 0.472, 0.973]) were significantly and clinically relevantly associated with decreased odds ratio for remission of depressiveness after psychotherapy. Pretreatment(s) (p = 0.061, OR = 0.403, [95% CI: 0.156, 1.041]) and education (p = 0.289, OR = 1.603, [95% CI: 0.670, 3.839]) may be considered clinically relevant predictors for remission of somatization. The explanation of variance at discharge was 15.9% for PHQ-9, and 8.4% for PHQ-15 in the adjusted model **(Table 3)**.

## Discussion

Predictors of symptom severity and successful inpatient psychotherapy are sparsely described. In this study, important sociodemographic and clinical factors were analyzed to fill this research gap. Particularly the role of the migration status regarding the severity of depressiveness and somatization at admission, and the success of psychotherapy of the respective symptoms was of interest.

In line with previous studies, belonging to first- or second-generation immigrants was a significant predictor for severity of somatic symptoms at baseline. Several studies have indicated higher prevalence or severity of somatoform symptoms/disorders among first- or second-generation immigrant patients [18, 27, 69–71], and also in non-clinical samples in comparison with reference samples of “autochthone” Germans [25]. There are several possible explanations for the stronger somatization tendency among first- or second-generation immigrant patients. Presumably pre-migration experiences (e.g., traumas) [72, 73] and postmigration stressors resulting from many significant losses (e.g., social status, social ties) and also the challenges associated with the acculturation process (e.g., learning a new language) [27, 74] or experienced discrimination may lead to a manifestation of somatic symptoms for distress [72]. More distance between the “host” culture and the culture of “origin” might account for more perceived discrimination, and therefore for more symptom burden [26]. It is known that different cultural models shape differently the experiencing of somatic symptoms and the reporting of such [75]. Migrants may express their distress in form of somatic symptoms to avoid stigmatization due to mental illness [76, 77]. Most probably the interaction of multiple factors involved, as the ones mentioned above, is responsible for the increased somatization tendency of first- or second-generation immigrant patients and not the migration status per se.

For a high level of somatization at baseline lower education, pretreatment(s) and belonging to first- or second-generation immigrants were significant negative predictors. In the first publication of this study we showed that first- or second-generation immigrant patients suffer significantly more from somatization, have significantly more somatoform-related diagnoses, and did not benefit significantly from psychotherapy concerning somatization in comparison to native German patients [60]. The fact that this study in addition detected belonging to first- or second-generation immigrants as a significant negative predictor for the severity of somatization at admission suggests that there is an important association between the migration status and somatization which must be examined more deeply in the future to address these patients adequately. Kirmayer stated that the widespread perception of “non-western” patients somatizing their psychological distress is obsolete, and that somatization is ubiquitous [78]. For the sake of all persons, it is important to ask all patients for somatization, and to understand it as a way of displaying distress.

For depressiveness at baseline, not living with partner, pretreatment(s), and migration status can be assumed to be potential clinically relevant predictors. There is evidence that a lack of social support and loneliness can be a risk for depression [79, 80]. Other studies have shown that for example a lower socioeconomic status could be significantly associated with higher depressive symptoms [81]. Since it is generally known that the socioeconomic status plays a crucial role in mental health, future studies should include the socioeconomic status in their survey [82]. Regarding the migration status as a potential clinically relevant predictor, it was demonstrated in earlier studies that similar to somatoform disorders, cross-culture differences also do exist for the features of depression, and that they are a function of the cultural shaping of normative and deviant behavior [83].

Cultural aspects such as different disease and health concepts [53, 84–86] play a very important role in mental health and in the way, patients react to psychotherapy. According to Franz et al. subjective illness perception is furthermore highly influenced by ethnicity [50]. Regarding the main cultural backgrounds represented in our investigation, studies show differences in mental health in comparison to non-immigrants or immigrants from other cultural backgrounds. Several studies from Germany demonstrated that in general Turkish immigrants suffer more often from mental illness or have higher symptom burden [15, 25, 69, 71]. Reich et al. showed that patients of Turkish origin tended to show higher fatalistic–external illness-related locus of control [53] which might be less compatible with the Western individualistic approach on psychotherapy. Concerning Polish and Italian immigrants, studies draw a similar picture in terms of poorer mental health compared to non-migrants [87–89]. In societies which are structured in a more collectivistic manner than a lot of Western societies, mental illness is often perceived as a psychosocial issue or an emotional reaction to disruption in social relationships [90]. Researchers and psychotherapists should therefore give more attention to the social surroundings and societal and cultural dynamics the individual patient comes from.

In contrast to former studies [15, 17, 20, 24] belonging to first- or second-generation immigrants did not prove to be a significant predictor for remission, neither in crude nor in adjusted regression models. However, taking into consideration a rather low odds ratio of the predictor migration status and the corresponding CI regarding the likelihood of remission of depression and somatization, it can be assumed that in a larger sample the effect of belonging to first- or second-generation immigrants on remission rates could probably be detected. Despite the non-significant p-values, clinical relevance can be postulated for belonging to first- or second-generation immigrants at least concerning remission of depression due to a relatively low odds ratio and the corresponding CI.

Most of the above-mentioned studies took place in rehabilitation settings or focused on special ethnic groups of migrants and are therefore not directly comparable with the results of our investigation. In these settings often migrants with insufficient language skills are treated. Yet, being this the first prospective study, examining the role of belonging to first- or second-generation immigrants on remission of depressiveness and somatization after psychotherapy, it is remarkable that the result from our study is contrary to former studies showing migration background to be a negative predictor for remission of somatization. This might be due to specific sociodemographic and migration-related characteristics of the migratory sample from our study. A large part of the migrants indicated at least good German language proficiency (50.9%) and most of first-generation immigrants had resided about 30 years in Germany [60]. Furthermore, no substantial differences were detected concerning important sociodemographic factors. Likely, most first- or second-generation immigrant patients from our sample can therefore be considered well integrated into the majority society and might bring along important resources for successful psychotherapy. However, on a societal level, large representative polls in Germany regularly demonstrate that these differences do exist regarding for example education, employment and income (i.e., educational degree: 84% native Germans vs. 64.8% first- or second-generation immigrants; average monthly net income 2225€ native Germans vs. 1869€ first- or second-generation immigrants) [7].

Regarding the complete sample, pretreatment and more diagnoses were associated with less odds for remission of depression. Also, higher education levels were related to relatively high odds and pretreatment(s) to relatively low odds for the remission of somatization. Therefore, based on effect sizes and corresponding CI clinical relevance regarding these predictors can be postulated despite non-significant p-values. For patients who have undergone several pretreatments or are diagnosed with several psychiatric diagnoses, reaching remission can be assumed to be more difficult.

## Strengths and limitations

This study is the first to prospectively examine important predictors, among them belonging to first- or second-generation immigrants, for severity at admission and remission after inpatient psychotherapy of depression and somatoform disorders in a clinical inpatient setting. We used psychometric instruments (PHQ-9 and PHQ-15) in order to measure the most frequent psychosomatic disorders, depression and somatoform disorders. Due to the wide reach of the clinics under investigation our study sample can be considered heterogeneous and relatively representative. The clinics are correspondent for rural as well as urban areas. The percentage of first- or second-generation immigrants was similar to the proportion in the general population (20.9% vs. 25.5% 2018 and 26.0% 2019). Similar to the largest migrant groups in the general population in Germany, Turkish and Polish origin were the most frequent ones among patients with a migration status. However, the small migrant sample made it impossible to carry out separate regression analysis for this group, or to detect small effects or predictors for subgroups within this heterogeneous sample (for example within different countries of origin or between first- and second- generation). Moreover, our results cannot be applied to the entire collective of immigrants living in Germany. Finally, the clinical diagnoses were not based on structured clinical interviews.

Our study sample might have a selection bias since only people with sufficient German knowledge were included. Presumably, people who suffer from mental diseases and cannot attend inpatient psychotherapy due to insufficient language skills might be more burdened and were not included in our study.

Topics of interest for further investigation in order to ascertain indicators for mental health among immigrants, should be for example, pre- (traumatic experiences) and post- migration stress (acculturative stress, perceived discrimination or consequences of low socio-economic status), as well as motivation and attitudes towards psychotherapy, or individual resources, such as coping strategies. Furthermore, the therapeutic relationship should be analyzed more deeply as it is crucial for successful psychotherapy. Possible individual or systematical barriers (such as perceived individual or structural disadvantages, personal uncertainties, or orientation difficulties in the German health care system) for a successful psychotherapeutic treatment of patients with migratory background should be investigated more deeply. To examine all these possible predictors of remission, it is recommendable to have a larger and more representative sample of different migrant populations in randomized controlled studies. A larger sample could also facilitate sufficient statistical power to test the measurement invariance of the scales across the groups. Previous studies examining measurement invariance of the questionnaires we used showed satisfactory results regarding language and ethnicity [91–93].

## Conclusions

The results of our study indicate an increased symptom severity in patients belonging to first- and second-generation immigrants at admission. Belonging to first- or second-generation immigrants seems to be a negative predictor of remission at least for depressive symptoms. Our findings suggest that belonging to first- or second-generation immigrants probably decreases the likelihood for remission. However, this finding needs to be verified in larger samples. Future research should investigate factors contributing to high symptom burden and successful treatments in first- or second-generation immigrant patients.
